# The Hospital Officer's Bookshelf

**Published:** 1913-01-04

**Authors:** 


### THE HOSPITAL OFFICER'S BOOKSHELF.

Aid to the Injured and Sick. By H. W. Gell, M.B. (The National Health Society. Thirty-seventh Edition. Pp. 40. Price 2d.)

Dr. Gell has certainly compressed the essentials of elementary first aid into smaller compass than most other authors who have essayed popular booklets of that sort. He has omitted surprisingly little, and his pamphlet well deserves its success. The published price of 2d. brings it within the means of the slenderest purses, and it is good to know that the standard of excellence reached is quite as high as that of many more expensive publications.

A Handbook of Nursing. By M. N. Oxford. Sixth Edition. (London: Methuen and Co., Ltd. 1912. Pp. 319. Price 3s. 6d.)

The choice of manuals on nursing is to-day a much wider one for the studiously inclined probationer than it used to be, but it is inevitable that for one reason or another the nurses at any particular hospital are generally inclined to favour one special text-book. Miss Oxford's book has now reached a sixth edition, and, apart from this, an examination of its intrinsic merits readily explains its popularity, not only at Guy's Hospital, but also among the nurses at many other hospitals. For the nurse a knowledge of the laws of life and health, of the functions of the various organs of the body, and of the outlines of its anatomy, is a distinct aid to her in the intelligent performance of her duties, and would leave her leisure to make herself proficient in those bedside duties on which depend the comfort and successful treatment of the patient. The study of such a text-book as this one will enable the nurse to obtain that necessary grounding which leads to the development of those qualities of observation and accurate reasoning so essential in the formation of a good nurse. Miss Oxford's text-book not only contains lectures on general medicine and surgery, but also chapters on some tropical diseases, monthly nursing, and the infectious fevers. Needless to say, the portion of the work dealing with nursing proper is characterised by that lucidity and practical method one knows is a feature of the teaching of the probationers of Guy's Hospital. There is a useful vocabulary at the end of the book.

The Care of European Children in the Tropics. By G. M. Harston, M.D. (London: Baillière, Tindall and Cox. 1912. Pp. 232. Price 7s. 6d. net.)

With the "conquest of the tropics"—not yet accomplished by any manner of means, but indisputably a mere matter of time—there is coming into prominence a revision of the old rules about the residence of European women and children therein. Far more wives now accompany their husbands to our tropical dependencies than ever before, and the consequence is that far more young European children are experiencing tropical conditions of life. In some regions—for instance, the highlands of British East Africa, which are quite equatorial—experience so far seems to show that white children can be successfully reared and grow up strong and healthy. In other regions the retention of children with their parents is only to be accomplished at the cost of incessant care and watchfulness. And in others it is still, as it used to be, practically impossible without the gravest risks to health and life. Dr. Harston, therefore, in devoting a monograph to the subject of tropical pædiatrics, is prospecting a rich and virgin field. It is not probable that all his views will meet with acceptance from every doctor with large tropical experience; but even his least orthodox opinions are so ably advanced and defended that it is a real pleasure to acknowledge the painstaking sincerity of his work all through. He has certainly earned the thanks of parents as well as of the medical profession, for though his pages are too technical for the former, they will of a surety prove an excellent clinical guide to the latter when called upon to advise concerning the ailments of white children in tropical countries.

The Baby. By A University Woman. (London: T. C. and E. C. Jack. Pp. 94. Price 6d.)

If the deluge of publications dealing with the care of infants is a sign of a steadily increasing public interest in the proper methods of rearing the next generation, not even the weariest reviewer can regret it. Possibly, too, the superabundant supply will create the demand; though this, we fear, is an economic heresy. At all events, the fact remains that a new book on this topic appears about once a fortnight. The newest has certainly an unusual touch about it, a personal tone which may recommend it to some of the mothers for whom it is designed. It is also short, concise, and cheap—three very great merits. It embodies the opinions and ideas of a "mother of three," who has evidently studied the question of infant care very thoroughly from a theoretical standpoint before putting principles to the test of practical experience. This domestic experience, at once limited in its scope and satisfactory in its results, has tended to narrow the author's views. We are quite confident she has been a most excellent mother to her own children; but also that if she had taken charge of a few score of other people's she would alter some of her opinions. By her own implication she knows nothing first-hand of the eruptive fevers and similar infantile diseases; yet she devotes more than a third of the book to their diagnosis and treatment. Unfortunately, her lack of experience has disturbed her sense of proportion in this section of the book. Thus she has evidently read in some text-book of the scarlatiniform prodromal rashes of smallpox; but these should find no place in her book. We find, too, that typhus fever receives quite inordinate attention: this and typhoid fever take up about 4½ per cent. of the whole volume. Smallpox, also, is dealt with at some length in two separate places. The University Woman would have produced a better book if she had collaborated with some experienced sister or matron of a children's or a lying-in hospital. Nevertheless, her book gives wonderful value for its price; and it is commendably free from mawkishness.

Another Device. By Stephen Paget, F.R.C.S. (London: Hodder and Stoughton. 1912. Price 5s. net.)

With certain very notable and brilliant exceptions the medical profession in bulk has always exhibited a poverty of literary expression and a lack of appreciation of the English tongue, for which there is little excuse. Lately this reproach has been to some degree mitigated, and it is to be hoped that in time a wider culture will diffuse itself among the ranks of the profession. Certainly there have always been shining examples of correct and graceful composition by medical authors, whether upon technical or general topics, and we incline to the belief that such examples have been less exceptional during this present century than at any former time. Amongst the literary artists of the day Mr. Paget already has an assured if modest position, and his English prose, even when it is most fiercely polemical and propagandist, is always a genuine source of pleasure for its accuracy and delicacy. In this new volume of essays the sureness of touch, the invincible honesty, the diligent pursuit of truth, and the essential kindliness and humanity of the author are all as obvious as ever. There is only one chapter on the controversial topics of which Mr. Paget is so able a protagonist; it is both trenchant and convincing (to our mind), as his utterances on "Christian Science" always are; but we could almost wish it had been omitted because it may give offence to estimable people who might otherwise find great pleasure in the book. We are bound to admit that Mr. Paget's courageous methods of tackling "Christian Science" and anti-vivisection, and all the fads of the day which are opposed to true progress and enlightenment, are necessary and salutary. But we think that, if he had omitted this particular essay, there would have been more chance of the faddists coming to recognise his sincerity and his logical processes of thought. All the essays are wholly delightful; two which appeal most to the present reviewer are those on "The Vale of White Horse" and on "The Right Sort of Girl." Anyone who can write such charming prose is fully justified in doing as Mr. Paget did when he gave up medicine to devote his energies to literature.

A System of Surgery. In Three Volumes. Edited by C. C. Choyce, B.Sc., M.D., F.R.C.S. Vol. II. (Cassell and Co., Ltd., London, New York, Toronto, and Melbourne. 1912. Price 21s. net.)

The generally favourable impression made on the reader by the first volume of Mr. Choyce's edition of the old Treves' "System of Surgery" is enhanced by a perusal of the second volume. This latter deals with the surgery of the breast, tongue and mouth, and gastro-intestinal and genito-urinary tracts. Like the first volume it is admirably printed and illustrated, and the student who purchases the work will not regret his outlay, for the book is an excellent compendium of English surgery. We notice, however, a certain discrepancy in the quality of the various sections. Where a compilation is the work of several individuals such discrepancies are only to be expected, for the guiding control of an editor cannot summon into line all his contributors, and produce a uniformity which is perhaps as undesirable as it is rare to find in books of this kind. Taking the second volume in detail, we find one of the junior colloborators, Mr. Ivor Back, contributing a section (that dealing with the salivary glands and the floor of the mouth) which for thoroughness of handling and conciseness is unequalled by any other chapter. Mr. Back deals with his subject in a thoroughly practical manner; he gives the student much information that is not usually to be found in text-books, and he has brought his nomenclature up to date and in agreement with that of Continental authorities, with whose work he appears to be thoroughly familiar. There is no partial bibliography appended to his article, but that is scarcely a fault, for, as we pointed out in reviewing the first volume, the bibliographical appendices are from the student's point of view superfluous and from the expert's too incomplete and unequal to be of any service. In the second volume this is even more apparent than in the first. By far the greater number of papers quoted are by well-known English and American writers, and here and there one notices glaring omissions. For example, Mr. Sampson Handley, who writes on the surgery of the breast and gives a concise epitome of his views as expressed in his larger work, omits all mention of Finsterer's monograph on carcinoma of the breast, which is one of the most instructive and complete synopses of the clinical and statistical evidence on this subject. Mr. Sherren's bibliography, the most ambitious in the volume, deals with the stomach and duodenum, but also omits important Continental authorities whose work is well known. His section is, however, a fair *résumé* of what the student should know concerning diseases of the stomach and duodenum and their surgery, although so important a point as dilatation of the stomach and its variations is rather scantily treated. Mr. Clogg deals with the rectum and anal canal, and his article at once invites comparison with its predecessor in the old system. So far at least as one point is concerned—hæmorrhoids—that comparison is distinctly in favour of the older volume. The clinical picture of a case of hæmorrhoids and anal fissure is by no means accurately described—the pain in a fissure case is usually described as following defæcation and increasing in severity after the act of expulsion, not culminating during the act—while the treatment of both important conditions is hardly described in such detail as it merits. Nor do we think Mr. Clogg has laid sufficient stress on the importance of examining the rectum in cases of pruritus; the most constant cause of this distressing condition is a small ulcer, and the application of soothing ointments is useless until the source of irritation is removed. Mr. Miles treats of the intestines; his section is clear and well illustrated, and some of his descriptions are excellent. Something more, however, he might have said about the initial signs of intussusception, particularly with regard to rectal examination in early cases. To wait for the usual signs in such a condition is to adopt a policy of procrastination which is not always advantageous to the patient. Mr. Sargent's contribution on appendicitis is admirably clear and its teaching is accurate, though here, too, it might have been pointed out that the signs of peritonitis are not to be taken as the guiding line in forming a diagnosis. Mr. McGavin deals with hernia; Mr. Rigby with the œsophagus. The latter appears to insist on a general anæsthetic in cases of œsophagoscopy; in our opinion the student should be taught to use the instrument on unanæsthetised patients with the aid of post-pharyngeal cocainisation. Mr. Nitch takes as his subject malformations of the face, lips, and palate, and deals with it in an able and lucid manner, though some of his illustrations are old and might with advantage be discarded for diagrammatic representations. As a matter of fact, the anatomical and embryological sections of the book are on the whole better done than those dealing with the symptomatology. Mr. Clayton Green writes about diseases of the tongue, and Mr. Grey Turner, of Newcastle, on those of the pancreas; while Mr. Walker and Mr. Howard deal with the urinary tract and the male genital organs respectively. Their several chapters are well done and should prove useful to the student. Mr. Bonney is responsible for an excellent gynæcological section, in which the pathology of the various diseases dealt with is admirably discussed, the illustrative woodcuts of microscopic sections being all that can reasonably be desired. A well-compiled index brings the volume to a close. While it is impossible to review the book in greater detail, this short abstract of its contents suffices to show its range and scope, and, although there are some points in each section to which a teacher may possibly enter objections, the general tone and handling of the various parts is surprisingly good.

